# End of OSLER Test Sessions in Parkinson’s Disease do not Correspond to True Sleep Onset: Results from an Exploratory Study

**DOI:** 10.3389/fneur.2015.00200

**Published:** 2015-09-24

**Authors:** Dulce Neutel, Rita Peralta, Joana Pires, Carla Bentes, Joaquim J. Ferreira

**Affiliations:** ^1^Department of Neuroscience, Centro Hospitalar Lisboa Norte, Hospital de Santa Maria, Lisbon, Portugal; ^2^Clinical Pharmacology Unit, Instituto de Medicina Molecular, Faculty of Medicine, University of Lisbon, Lisbon, Portugal; ^3^EEG/Sleep Laboratory, Department of Neuroscience, Centro Hospitalar Lisboa Norte, Hospital de Santa Maria, Lisbon, Portugal; ^4^Laboratory of Clinical Pharmacology, Faculty of Medicine, University of Lisbon, Lisbon, Portugal

**Keywords:** sleepiness, sleep onset, OSLER test, Parkinson’s disease

## Abstract

The aim of the present study was to evaluate the correlation between the end of an Oxford sleep resistance (OSLER) test session and a neurophysiological marker of sleep onset in Parkinson’s disease (PD) patients. Single center study was conducted in PD patients with excessive daytime sleepiness [Epworth sleepiness scale (ESS) >9]. The OSLER test was conducted with a concomitant electroencephalography (EEG), electromyography (mentalis), right and left electroculogram, and video monitoring. Neurophysiological (NP) sleep onset was defined according to AASM criteria (2005). Five PD patients with mean ESS of 14 (10–16) were included. OSLER test duration was shorter than 40 min in all patients (mean duration 20 min and 39 s). No patient fulfilled neurophysiological criteria to sleep onset at the time of OSLER test termination. In 13 OSLER sessions that ended before 40 min, eight had microsleeps in the last 30 s before the end of the test. NP monitoring showed signs of sleepiness in all patients. In PD patients, the early termination of an OSLER test session may not correspond to NP criteria of sleep onset. However, in all PD patients with abnormal OSLER results, there were EEG signs of sleepiness, which do not exclude the potential utility of OSLER test to evaluate the risk of falling asleep.

## Introduction

It is well established that excessive daytime sleepiness (EDS) in Parkinson’s disease (PD) significantly interferes with the patient’s quality of life and constitutes an important risk factor for accidents (1, 2). In clinical practice, the quantification of EDS and its relation, for instance, to driving fitness is a critical issue and physicians increasingly need to screen patients at risk of sleep-related accidents. However, the assessment of EDS is complex; complaints do not always coincide with an objective impairment of vigilance (3), and patients often do not recognize they suffer the problem, which increases largely several risks as those related to driving (4).

Several sleep scales have been used to evaluate PD sleep disorders, but only a small number has been assessed specifically in a PD population (3, 5–13). The International Parkinson and Movement Disorder Society recommends the Epworth sleepiness scale (ESS) (5) for use in PD for daytime sleepiness based on a review of the validity and clinimetric characteristics of all available sleep scales that have been applied to PD patients (13). Importantly, sleepiness scales as the ESS or Stanford sleepiness scale (SSS) (6) do not strongly correlate with objective quantifications (14). Furthermore, specific PD-related problems, such as the occurrence of motor symptoms and fluctuations, fatigue, cognitive impairment, depression, and medication side effects, may interfere with the validity of generic sleep scales.

Assessing daytime impairment by using objective quantification tools may provide a better evaluation of daytime function changes in these patients. The standard objective tests of daytime sleepiness are the multiple sleep latency test (MSLT) (14, 15) and the maintenance of wakefulness test (MWT) (16). The MSLT and MWT provide quantitative measures of daytime sleepiness and ability to maintain vigilance. Both tests are based on latency to electroencephalographic sleep onset. However, the need for daytime electroencephalography (EEG) monitoring and technician attendance renders these tests labor intensive and expensive.

To overcome these difficulties, Bennett and Davies (17) proposed a behavioral test, called the Oxford sleep resistance (OSLER) test, which utilizes a computerized, non-assisted method for monitoring wakefulness and detecting sleep onset. However, failure or inability to cooperate can result in false positive results in OSLER test. False positives may occur due to a patient’s lack of attention, fatigue, motor impairment, and frequent eye blinking. One way to overcome this limitation is to evaluate with a concomitant neurophysiological study the moment that patients stop pressing the button and verify if it corresponds to a true sleep onset. The main objective of this study was to document if the early end of an OSLER session in PD patients corresponds to true sleep onset measured with neurophysiological recordings and to support its validity as a measure to analyze the tendency to fall asleep. A secondary objective was to verify if early terminations were associated with clinical findings or other sleep-related EEG.

## Materials and Methods

### Patients

A convenience sample of five PD patients with ≥3 years of motor symptoms and complaints of EDS were recruited from the Movement Disorders Unit of the Lisbon University Hospital. Eligible patients had a diagnosis of idiopathic PD (18), an ESS (5) score ≥10, and (in the investigators judgment) the capacity to perform the evaluation tests. Non-eligibility criteria were Mini Mental State Examination (MMSE) score <24 (19), clinical diagnosis of depression or active psychosis, severe dyskinesia (UPDRS 32 > 1 or 32 > 1 in ON), treatment with hypnotics or psychotropics with known sedative effects, or suffering from other medical diseases known to cause daytime sleepiness (e.g., obstructive sleep apnea or periodic limb movement disorder). The presence of fatigue was screened for using the 9-item fatigue severity scale (FSS) (20, 21) and the presence of apathy was diagnosed according to Starkstein and Leentjens diagnostic criteria (22–24) and quantified using the 14-item Apathy Scale (AS). All participants signed an agreement to take part in the study. The study was approved by the local ethics committee.

### Study design

This study was conducted in a single center. Patients underwent the usual OSLER protocol with four periods of a maximum of 40 min OSLER session with an interval of 2 h in between. Patients were instructed not to sleep between sessions and not to consume alcohol, caffeine, chocolate, tea, hypnotics, or benzodiazepines for 12 h before the evaluation day and for the duration of the evaluation procedures. A full night polysomnography (PSG) was performed to exclude other sleep disorders known to cause daytime sleepiness (according to American Sleep Disorders Association criteria) (25).

### Measurements

#### OSLER Test

The OSLER test consists of a 40-min sleep-resistance challenge conducted in a dark and quiet room (17). It features a small light-emitting diode (LED) in a wall-mounted box – and a patient-held pressure-sensitive switch box, both attached to a control unit connected to a computer. Lying down in a darkened room, patients were asked to press the switch in response to the illumination of the LED, which was set to occur every 3 s. The switch had no audible click. Patients were asked to stay awake but not to use active methods to keep themselves awake, such as singing, shouting, or pinching themselves. A software program controlled the LED and stored the responses. “Sleep onset” was defined as missing seven presses, equivalent to 21 s of no response. The investigator remained in the same darkened room as the patients, in order to clinically monitor their behavior. Patients underwent an OSLER test at 9:00 a.m., 11:00 a.m., 1:00 p.m., and 3:00 p.m. and the session was terminated after sleep onset was determined by the OSLER or after 40 min, whichever came first. The total score was defined as the mean time to “sleep onset” over the four periods.

#### Electroencephalography

Electroencephalography was monitored during OSLER test using F3/A2, F4/A1, C3/A2, C4/A1, O1/A2, O2/A1, two horizontal electrooculographic channels (EOG R e L), and submental electromyography (EMG). Neurophysiological sleep onset was defined as the first epoch of 30 s scored as any stage other than stage W (wakefulness) and stage N1 as alpha EEG waves replaced by theta-frequency (4–7 Hz) waves that occupy >50% of the epoch; if alpha waves are not readily apparent, the presence of 4–7 Hz waves with slowing of background activity by at least 1 Hz compared with stage W, vertex sharp waves, or slow eye movements also can be used to document the start of stage N1 (American Academy of Sleep Medicine). Microsleeps were defined as a period of at least 3 s with a theta (4–7 Hz) rhythm replacing an alpha rhythm or appearing on a background of desynchronized EEG on all EEG channels, and without eye-blinking artifacts. Slow eye movements were accepted. No maximal duration was defined for microsleep, so that it could evolve into established sleep if it lasted long enough.

#### Other Evaluations

The self-rated SSS was used for rating sleepiness and to measure its severity just before each OSLER test (6). The SSS is a seven-point Likert-type scale where a high score indicates a high level of sleepiness and patients were instructed to choose the set of descriptors that best described his or her current feeling of sleepiness just before each OSLER test.

In addition, on the day of the OSLER test with EEG records, all patients were evaluated using the MDS-UPDRS (26) and the Hoehn and Yahr (H&Y) scale (27) while in the ON-state.

### Data analysis

Data were analyzed using descriptive statistics and will be expressed as the median or mean ± SD.

## Results

### Patients clinical characteristics

Patients (median age 62, range 56–72) included in this study had a median PD duration of 4 years (range 3–7 years) and demonstrated a broad range in UPDRS scores (total) (39–76). Screening for fatigue and apathy found only one patient who met diagnostic criteria for both (Patient 2). Individual patient data and means are shown in Table 1.

**Table 1:** **Clinical characteristics of patients**.

		1	2	3	4	5	Mean
Gender		Male	Male	Male	Male	Male	
Age (years)		62	72	56	64	58	62.4
PD duration (years)		3	4	10	4	7	5.6
UPDRS	Total (I + II + III + IV)	39	76	50	47	56	53.6
	Part III	26	49	36	31	32	34.8
Hoehn and Yahr		2	2	2	2	2	2
Epworth SS		10	16	15	14	15	14
Standorf SS		1	1	1	2	2	1.4
Apathy SS		34	43	35	33	38	36.6
Fatigue SS		20	53	21	23	23	28

### Polysomnography

The full night ambulatory PSG performed in the night before the OSLER test showed mild OSAS in one patient. No other relevant respiratory disturbances or periodic limb movements were found in the other patients (Table 2).

**Table 2:** **Ambulatory polysomnography results**.

Patients	PSG				
	AHI	PLMs	ODI	Hypnogram	Conclusion
1	0.9	0	5.1	↓ Sleep efficiency + sleep maintenance insomnia + ↓ REM	Without OSAS or PLMDs
2	3.2	0	12.1	↓ Sleep efficiency + initial and sleep maintenance insomnia	Without OSAS or PLMDs
3	10.4	0	15.1	Sleep maintenance insomnia	Mild OSAS
4	0.3	2.9	10.4	↓ Sleep efficiency + ↑ awakenings	Without OSAS or PLMDs
5	2.6	2.8	7.8	↓ Sleep efficiency + sleep maintenance insomnia	Without OSAS or PLMDs

*PSG, polysomnography; AHI, apnea/hypopnea index; PLMs, periodic limb movements; ODI, oxygen desaturation index; OSAS, obstructive sleep apnea syndrome; PLMDs, periodic limb movement disorders*.

### OSLER test results

The OSLER test duration was shorter than 40 min in all the five patients. However, no patient fulfilled neurophysiological criteria to document sleep onset at the time of OSLER test termination. Only one patient fell asleep while performing the OSLER test. In this patient, there was waxing and waning alpha activity, fulfilling the criteria to score N1 sleep stage even before the OSLER test termination. Table 3 compares the sleep latencies of individual sessions as defined by the OSLER test and the simultaneously recorded EEG.

**Table 3:** **OSLER tests results with simultaneous neurophysiological records**.

Patients	OSLER test (minutes)		EEG	
	Mean sleep latency (normal 39.8 min)	Sleep latency	During all OSLER test	Last 30 sec. of OSLER test
1	00:25:50	00:39:09	Alert	Wakefulness
		00:01:24	Alert	Wakefulness
		00:22:48	Alert	Wakefulness
		00:40:00	Alert	Wakefulness
2	00:04:06	00:02:09	00:00:08 (N1)	Microsleep
		00:01:21	00:00:10 (N1)	Microsleep
		00:05:27	00:00:41 (N1)	Microsleep
		00:07:27	00:00:14 (N1)	Microsleep
3	00:36:37	00:26:27	Alert	No EEG record
		00:40:00	Alert	Wakefulness
		00:40:00	Alert	Wakefulness
		00:40:00	Alert	Wakefulness
4	00:23:29	00:03:51	Alert	Microsleep
		00:40:00	Alert	Wakefulness
		00:10:03	Alert	Wakefulness
		00:40:00	Alert	Wakefulness
5	00:13:12	00:16:33	Alert	Microsleep
		00:03:54	Alert	Microsleep
		00:07:54	Alert	Microsleep
		00:24:27	Alert	Wakefulness

*EEG, electroencephalogram; sec., seconds; N1, N1 stage of NREM sleep*.

Although none of the patients fulfilled neurophysiological criteria of sleep onset in the end of OSLER test, we found that in 13 of the 19 OSLER sessions that ended before 40 min, 8 had microsleeps in the last 30 s before the end of OSLER test (Table 4). Furthermore, neurophysiological monitoring showed other markers of sleepiness in all patients, such as slow eye movements and increased blinking. One patient performed significant periods of the test with their eyes closed.

**Table 4:** **Sensitivity of OSLER test for microsleeps**.

	Microsleep (+)	Microsleep (–)	
OSLER (+)	8	5	13
OSLER (−)	0	6	6
	8	11	19

*OSLER (+), OSLER test ended before 40 min; Microsleep (+), microsleep in the last 30 s of the OSLER test*.

*Sensitivity of OSLER test for Microsleeps – 100%; specificity of OSLER test for Microsleeps – 55%; positive predictive value – 62%; negative predictive value – 100%*.

## Discussion

Theoretically, the moment when patients stop pressing the switch for 21 s is supposed to represent sleep onset, and previous studies in non-PD populations have found that the OSLER test is capable of discriminating between sleepy and normal individuals without overlap (mean latency 10.5 and 39.8 min, respectively) (17). We have previously reported a study in which all sleepy PD patients had an abnormal OSLER sleep latency (28). Now, we found no correspondence between the end of the OSLER test sessions and the documentation of true neurophysiological criteria for sleep onset.

Nevertheless, all five sleepy PD patients had OSLER sleep latencies of <40 min. Although we did not have a control group, previous studies in the literature show that this finding is abnormal. In the studies conducted by Bennett (17) and Mazza (29), all control subjects were able to finish the 40-min test without falling asleep. If we apply another commonly used cut-off score for abnormality (20 min) (30), two of the five patients presented abnormal scores in the OSLER test.

The neurophysiological criteria for sleep used in this study, are the same that are used in MSLT and MWT, and requires the presence of theta rhythm in at least 50% of a 30 s epoch (that it, at least 15 s). These criteria have low sensitivity for sleepiness: sleepiness begins even before reaching stage 1 NREM sleep. Furthermore, there is no exact moment of sleep onset – there are gradual changes in behavior, reaction time, and cognitive function and physiological changes. This stage has been coined as the “Pre-dormitum” (31). It is defined as having behavioral signs such as heaviness and drooping of the eyelids, clouding of the sensorium, and inability to see, hear or perceive things in a rational or logical manner. Therefore, the detection of microsleep has been described as a better measure of sleepiness (32). Indeed, our study found that 62% of the OSLER sessions with durations below 40 min had microsleeps and the OSLER test showed a sensitivity and a negative predictive value of 100% for microsleeps (Table 4). Furthermore, all patients had neurophysiological (slow eye movements, increased blinking rate) and behavioral sleepiness. Taken together, this suggests that OSLER test termination in our PD patients is mainly caused by sleepiness.

In their cross-sectional sleep laboratory study (*n* = 11), Krieger and colleagues (30) verified that a sleep latency by OSLER test and simultaneous measurement of EEG had excellent agreement (ICC = 0.91) with a bias of – 0.97 min (30) when sleep was defined as episodes of sleep of ≥3 s duration, similarly to our microsleep criteria. Therefore, OSLER test results in our PD population appear to be less specific to detected microsleeps than in other populations. In their study conducted in normal subjects, Priest et al. (33) reported that only 2 of 37 OSLER test terminations were not associated with microsleeps. The authors attributed these results to lapses of attention and misunderstanding of the test’s instructions. While the latter is unlikely in our patients (all patients received detailed instructions prior to the tests), lapses of attention are a possible contributing factor. It is noteworthy that some of the patients showed performed parts of the OSLER test while showing alpha rhythm in the EEG. Alpha bursts during active tasks have been associated with attention lapses (34). In addition, the higher number of false positive results in PD patients could be related to disease specificity; for example, visual processing deficits, frequent and slow eye blinking, or motor impairment that leads to finger motion below levels detectable by the OSLER system could have impacted on the test results. Although only one of our patients met the criteria for apathy and fatigue, these symptoms were not directly assessed at the time of the OSLER session and cannot be ruled out. It should be noted that the only patient who met criteria for fatigue, apathy, and had also the worst motor performance, and was the only patient who actually fell asleep (EEG criteria) during OSLER test.

The patient who fell asleep according to neurophysiological criteria did it in the first minute of the test. However, he was able to perform the OSLER test for a mean duration of 4 min and 6 s. This is in line with other studies and indicates that automatic behavior is still possible in the transition from wakefulness to sleep (35). It is important to note that the OSLER test is only finished when seven consecutive presses are missed. In the study of Priest et al. (33), 25 out of 57 epochs of sleep (defined as a microsleep with more than 15 s) led to 2–6 consecutive missed responses therefore allowing the test to continue. The authors of that study suggested that the inclusion of the error rate during the test might increase the sensitivity of the OSLER test to detect microsleeps, as the possibility to detect microsleeps increases in parallel with the number of consecutive lapses. This approach as been later replicated in a study of sleepy OSAS patients and controls and may be useful to try in future PD studies (29).

We used ESS for screening for sleepiness in PD patients as recommended by the International Parkinson and Movement Disorder Society but it has recognized limitations. Some studies using ESS have shown best correlation for pathological daytime sleepiness with a higher cut-off point (>14). So, it will be important to look for different results using different cut-off points in future studies using OSLER test (36). Also, although we used video recording simultaneously to OSLER test for evaluation of motor performance it was not possible to rule out with absolute certainty all the motor phenomena that could compromise test performance. Additionally, it would have been useful to compare the OSLER results with MWT in order to validate OSLER test as a measure to resist sleep. Finally, we should emphasize that the PD population often has transient abnormalities in the baseline EEG (notably slow activity, like theta bursts), which are common and do not necessarily mean sleepiness. It is not entirely possible to rule out that the microsleeps scored in this study are abnormal EEG transients. However, the concomitant presence of other markers of sleepiness (slow eye movements, increased blinking) (37, 38) in our patients supports our assumptions that the theta burst scored are in fact microsleeps.

In conclusion, the results of this small study show that the OSLER test in PD patients does not measure true time to sleep onset. However, it seems to be a sensitive tool to detect microsleeps in PD. Further studies are needed to find out the clinical meaningfulness and usefulness of OSLER test in the determination of the tendency to fall asleep in patients with Parkinson disease.

## Conflict of Interest Statement

The authors declare that the research was conducted in the absence of any commercial or financial relationships that could be construed as a potential conflict of interest.
